# LONGITUDINAL CARE ARRANGEMENT PATTERNS AND SUBJECTIVE WELL-BEING AMONG COMMUNITY-DWELLING OLDER ADULTS

**DOI:** 10.1093/geroni/igae098.3391

**Published:** 2024-12-31

**Authors:** Esther Shin, BoRin Kim, Sojung Park

**Affiliations:** Illinois State University, Normal, Illinois, United States; University of New Hampshire, Durham, New Hampshire, United States; Washington University in St. Louis, St. Louis, Missouri, United States

## Abstract

Many older adults with health problems receive assistance from caregivers in their communities. The availability and nature of caregiving (e.g., formal or informal) is contingent on varying individual and contextual factors with important consequences in the care recipients’ life quality. Existing research tends to examine single sources of caregiving. Even when examined together, the majority of current investigations focus on older adults with dementia. Using the National Health and Aging Trends Study (2011-2022), this study identified distinct longitudinal trajectories of informal and formal caregiving and their association with subjective well-being among community-dwelling older adults aged over 70. We restricted our sample to those with caregiver support throughout the study period (n=1,200). Using group-based multiple trajectory modeling, we identified four longitudinal care patterns: (1) Consistently limited care (9.3%), (2) Predominantly sole informal caregiver (57.5%), (3) Multiple informal caregivers (17.2%), and (4) Dual caregivers (16%). In the consistently limited care group, older adults were more likely unmarried, educated, non-white, living alone, and with mobility issues. Those with multiple informal caregivers tended to be older, female, in poor health, and living in independent/assisted living. Conversely, older adults in dual caregiving were likely educated, covered by Medicaid, and residing in retirement communities. Findings highlight the role of sociodemographic factors in shaping patterns of caregiving arrangements, and having dual caregiving was associated with increased well-being among care recipients. Creating an affordable care scheme would be essential in supporting healthy aging among older adults who wish to remain at home with adequate caregiving support.

